# What Young People Think About Music, Rhythm and Trauma: An Action Research Study

**DOI:** 10.3389/fpsyg.2022.905418

**Published:** 2022-06-14

**Authors:** Katrina McFerran, Alex Crooke, Zoe Kalenderidis, Helen Stokes, Kate Teggelove

**Affiliations:** ^1^Faculty of Fine Arts and Music, The University of Melbourne, Melbourne, VIC, Australia; ^2^Melbourne Graduate School of Education, The University of Melbourne, Melbourne, VIC, Australia

**Keywords:** rhythm, trauma, music therapy, action research, co-regulation, arousal

## Abstract

A number of popular theories about trauma have suggested rhythm has potential as a mechanism for regulating arousal levels. However, there is very little literature examining this proposal from the perspective of the young people who might benefit. This action research project addresses this gap by collaborating with four groups of children in the out-of-home-care system to discover what they wanted from music therapists who brought a strong focus on rhythm-based activities. The four music therapy groups took place over a 12 month period and each cycle of action and reflection led to adjustments in what activities were offered, as well as exploring different levels of structure and ways of building relationships in the groups. The initial group incorporated a strong emphasis on highly structured rhythm-based activities, but young people found the format difficult to engage with. The second cycle included more opportunities for creativity and self-direction within semi-structured activities which children reported enjoying, but too much freedom also became overwhelming at times. The two groups in the third cycle seemed to balance structure and responsiveness successfully but were also influenced by the introduction of individual sessions prior to group commencement, which was designed to contribute to safety and trust building. Final reflections on the role of rhythm in supporting young people who have had adverse experiences were centred around the ideas of co-regulation. This was qualitatively different to our expectations that practicing rhythm-based activities would lead to an expanded window of tolerance that resulted in less time being spent in either hypo-arousal or hyper-arousal. Instead of entraining to an external rhythm, young people felt safe when their rhythms were matched, even if they were irregular, out of time and unpredictable. The small moments of co-regulation resulted in pleasure, comfort, satisfaction and peace and these moments were highly valued by the young people, who described just wanting to be relaxed and happy. Although not as rhythm-specific as the literature might suggest, music making with trusted adults helped the young people in this study feel more content.

## Introduction

The discourse on trauma recovery is currently dominated by neurological models. Two of the most popular theorists quoted in this discourse have suggested that rhythm has potential as a mechanism for regulating arousal levels. This includes Porges’s (2011) emphasis on regulating a hyper-vigilant amygdala and van der Kolk’s (2015) focus on stimulating positive and reparative experiences through repetitive neural brainstem activity. The emphasis in both these theories is on engaging primitive neurological functions and both theorists adopt a medicalised view of trauma that can be resolved by repairing damaged areas of the brain.

This contrasts starkly with older, psychoanalytic models that focussed on processing experiences through the resolution of discord between conscious and unconscious awareness (Brunner, 2018). This approach to the resolution of trauma became prominent after the recognition of a link between behaviour labelled as “hysteria” and repressed memories of adverse experiences. These ideas were developed by French theorists around the turn of the century, firstly by Jean Martin-Charcot and then Pierre Janet through understanding the mechanism of dissociation (Walusinski and Bogousslavsky, 2020). Sigmund Freud is also recognised for his contributions in the Austrian context, although interpreted differently through the case study of Dora (Freud, 1953). Music (but not specifically rhythm) has been utilised in psychoanalytic approaches because of the affordances of improvised music as a “blank page” onto which unconscious meaning can be externalised and made apparent (Priestley, 1994). The recent interest in rhythm might be seen to have renewed interest in the role of music in therapy with people who have had adverse life experiences (Hillman et al., 2021).

It is noteworthy that both the older and the new perspective have been established by privileged white men in developed societies primarily for the purpose of treating the problems of abused women and children. This requires critical understandings be considered, particularly examining whose voices are being ignored while others are privileged. The most outstanding exception to the privileged standpoints to date has been Judith Herman’s (1997/2015) work on trauma and recovery, in which she introduced a systems lens to conceptualisations of trauma in the United States in the 1990s. Herman emphasises more contextualised and idiosyncratic understandings of people’s unique responses to a range of adverse experiences and does not propose solutions, but rather ways of understanding. Music therapists frequently reference Herman’s work (McFerran et al., 2020), as do educators (Stokes and Turnbull, 2016), and the emphasis in programs that align with her model are usually on building trusting relationships and the importance of safety, in education.

In considering whose voice is missing then, one significant group is the young people who have been traumatised by adverse experiences and whose perspectives have been sparsely represented in the psychiatric literature, although are more often included in education discourse (Stokes et al., 2019; Stokes and Aaltonen, 2021). Their absence is noteworthy in the music therapy literature (Lai et al., 2020) and although many authors emphasise the importance of trusting relationships and experiences of safe attachment, this has traditionally been presented from the perspectives of the adult practitioners. There are ethical reasons for this. Knowing young people have often been at the disposal of adult needs and expectations through their adverse experiences, many of us have strong urges to protect them from further demands. The ethical research systems are also designed to protect children in this way, and the national ethics guidelines specifically identify challenges for children’s understanding of what they are agreeing to and vulnerability to coercion (NHMRC, 2018 Updated 2018). However, this can also lead to a lack of representation. The Australian guidelines acknowledge this, and further state that “there are particular tensions between not placing children at risk in studies of new interventions and the need for knowledge about how such interventions are best used for children.” (p. 65). If we assume that young people are too vulnerable to risk engaging in the intellectual field that shapes the support they are surrounded by, then we also disenfranchise their perspectives.

Even if we overcome the ethical barriers of gathering the perspectives of young people who have had adverse experiences, we also face some cognitive challenges. Young people who have experienced neglect often do not have the vocabulary to articulate the complexity of their experience, sometimes described as alexithymia (Terock et al., 2020). According to cognitive developmental theories based on Jean Piaget’s work (now critiqued, Babakr et al., 2019), young people also do not have the cognitive ability to give more than an egocentric response, when research questions are seeking a more meta-perspective. When using music, this challenge of articulation is intensified, since even professional adults struggle to find words to describe the embodied experience of shared music making for therapeutic benefit. This is seen by the emphasis in the literature on case studies and investigations of professional experiences where rich descriptions of practice are elaborated. The use of case studies seems to be the closest researchers feel able to come to describing the phenomenon. Again, the lack of representation is partly explained by the challenge of collecting the alternate perspectives from young people.

Our intention in this research study was to critically examine the ideas in the trauma literature that suggested rhythm might have unique affordances for processing and resolving trauma. We acknowledge how attractive theories on rhythm are in the field because they can be used to justify existing music-based practices and provide a scientific explanation for mechanisms that are otherwise difficult to articulate. However, the assumption that playing in rhythm is helpful was not a good match with our practice-based experiences of working with youth with experiences of trauma who may not have musical training and who find steady rhythms both challenging and disheartening. We also questioned the move away from responsive and relational program planning and toward highly structured and pre-determined activities that were congruent with a brain-based approach where rhythm had a healing influence (Colegrove et al., 2018), and is more similar to models such as EDMR (Oren and Solomon, 2012). We wanted to understand when rhythm was experienced as helpful by young people and to explore if there were identifiable conditions associated with those moments. We decided to focus on young people’s actions, and to explore different rhythm-based activities with different groups of young people in iterative cycles that enabled co-construction of knowledge. We aimed to adjust and adapt to what young people seemed to want, both within each session and structurally between sessions and between programs, and listen to what was expressed by the young people through their engagement, their artistic expression and their words. We endeavoured to answer the question:

How satisfied are young people in the out-of-home-care system who participated in various rhythm-based music therapy activities, as communicated by their verbal and written feedback as well as their observed responses and actions?

## Methodology

Despite the dominance of highly intellectualised neurological theories in research in the trauma field, we chose to privilege experiential knowing as potentially leading to novel discoveries that have greater relevance for practice. Given the effectiveness of play-based methods with marginalised young people (Post et al., 2019), we selected research methods that enabled multiple ways of communicating how satisfied the young people were with the programs. By integrating non-verbal feedback, we were able to draw on our practice experiences as music therapists who grapple regularly with the challenges of interpreting arts-based and behavioural responses as an equally interesting way of understanding phenomena (Chamberlain et al., 2018). This interpretation is the basis of creative arts therapies generally and the reason it is often posed as an alternative to talk-based therapies when people have experienced adversity (e.g., Fairchild and McFerran, 2019). Arts-based research has recently gained prominence in the field, with theorists arguing that the sensitivity of the arts enables understandings that embrace emotional aspects as well as ambiguity and paradox, which may be relevant in this kind of research (Leavy, 2020). Aesthetic theories (Juslin, 2013) and musical theorising (Ferrara, 1991) already exist that can be used for analytic meaning-making purposes and have been in understanding existential crises such as grief and loss (McFerran and Wigram, 2005).

Action research design supports this approach, with an emphasis on both situated processes of collaboration and extended notions of knowledge (Greenwood and Levin, 2006). Polanyi’s (1958/1998) valuing of personal knowledge and moving beyond propositional theorising has influenced many action researchers who have instead emphasised presentational and relational ways of knowing (Smith et al., 1997). In the field of music therapy, action research has often been used for examining community-based programs with people who are marginalised (Stige and McFerran, 2016), and the combination of arts-based approaches and action research values have been emphasised in some approaches where the arts might be either a methodology, or a radical event (Ledger and McCaffrey, 2015). Action research is also commonly used for the purpose of planned change and improvement (Cohen et al., 2007; Kemmis et al., 2014). The reflective and cyclic nature of action research provides researchers with opportunities to ascertain the degree to which the intervention is a success and to analyse the data for why this might be the case. Following this they can then proceed or make necessary changes to the intervention.

## Actions and Reflections Within the Program

### Approval and Collaboration

We were granted ethics approval from the university ethics committee (ID#1852103.1) to undertake an action research project in partnership with a not-for-profit community provider who work with children, young people and families to prevent harm and empower families including home-based care (foster care), parenting support, and family violence programs. The provider was responsible for recruitment, using materials that we generated, and which advertised “a fun, music-making group for young people (from their service) aged 8–12.” They also made rooms available, liaised with other community providers and were supportive of our desires to understand whether music therapy was useful for children in their care. They benefitted from the provision of fee-free programs they could promote to their clientele without monetary investment. An internal research institute at the university provided the funds for the investigation, with the requirement that community collaboration be centralised in the research and that youth voice be prominent in as many aspects of the design as possible.

### Exploring Structure for the Program

There were three cycles of action and reflection across a 12-month period involving a total of 16 young people (see Table 1). Two qualified music therapists co-facilitated groups of 3–5 members, lasting between 8 and 13 weeks, supported by a community facilitator who was also one of the researchers. Group size was intended to include a maximum of 6 young people, but the number of cycles was not pre-determined, except by the 2-year time frame of research funding.

**TABLE 1:** Overview of research process.

	Cycle 1	Cycle 2	Cycle 3	
Program	1	2	3	4
Length	13 weeks	8 weeks	8 weeks	8 weeks
Youth	5 young people	3 young people	4 young people (1 withdrawal/child relocated after week 3)	4 young people
Referral	Experienced trauma and displaying aggression at school, observations of being withdrawn/emotional, needing extensive support with regulation, complex parent mental illness and parent death (witnessed).	Experienced trauma with reports of experiences of ableism/restrictive practice at mainstream school (resulting in home schooling), witnessing domestic violence and removal from home at a young age. Two young people lived with immediate family and one had experienced many care placements.	Difficulty regulating emotions, swings in mood, barriers in accessing school. One young person lived with immediate family, two were in kinship care and one was in permanent care.	Lived experience of homelessness, needing extensive support with regulation, complex parent mental illness. Two young people were living with immediate family and two were in permanent care.
Session structure	Group sessions only. Pre-set session plan delivered with no (or very limited) choice for participants	Individual sessions provided before group commencement. Pre-set session plan with options for choice of one or two activity.	Individual sessions provided before group. Session plans negotiated from set options with young people.	
Rhythm-based activities	Tune in sheet Ti Rakau Obwisana Rock Games Beanbag Toss Rapping/Songwriting Dance off Djembe Drumming	Tune in sheet Ti Rakau Obwisana Rock Games Beanbag Toss Rapping/Songwriting Dance off Djembe Drumming	Tune in sheet Ti Rakau Beanbag Toss Rapping/Songwriting Dance off Djembe Drumming Chair drumming	
Evaluation strategies	SERQ and DERS measures Semi structured interviews Parent/carer feedback	6 item self-designed scale Semi structured interviews Parent/carer feedback	Prioritised incidental feedback. Arts-based feedback activities Semi structured interviews Parent/carer feedback	

As summarised in Table 1, the content of the program evolved in response to the feedback from young people. Their feedback was shared *via* their behaviour, levels of engagement, comments made during sessions and at closure of the sessions, as well as from comments made by carers. Our understanding of their feedback directly influenced what activities were prominent in sessions and what evaluation measures we used to try to capture any benefits experiences using objective measures. The details of this process will now be described.

### Measuring Responses

During Cycle 1, we discovered that the young people were not able to complete the measures we had identified as being most suitable to capture any changes in self-regulation that might be experienced because of participation [Social Emotional Competence Questionnaire (Zhou and Ee, 2012) and the DERS-16 (Bjureberg et al., 2016)]. When presented with the forms they expressed disappointment in having to do what felt like “work,” fell into patterns of ticking consecutive boxes rather than contemplating authentic responses, and engaged in critical rumination about behaviours they believed to be negative or bad. Varied literacy levels caused embarrassment and defensive responses from some and it was clear that the ideas being assessed were too complex.

Therefore, in Cycle 2 we moved to session evaluation strategies that used a shorter six-item self-composed measure designed to capture the differences between the young people’s baseline state at the commencement of the program (reflecting on life in the 2 weeks prior) and how they felt as each session ended. This tick box measure was created with specific reference to factors stated in interviews with participants in Cycle 1 as being experienced within sessions including safety, belonging, freedom, pride and self-control (See Appendix 1). A mid program “check-in” was also introduced to enable participants to offer feedback on what was helpful and preferred in sessions, aimed at determining session content for the remainder of the program.

By Cycle 3, we understood through verbal and behavioural feedback from the young people that written evaluations and measures were generally not well accepted or effective in capturing or understanding participant experience. Even the short, simple questions elicited defensive, disengaged behaviours as seen by crumpling the paper into a ball or using it for drawing unrelated sketches by memory or leaving the table to attend to other things in the room or even just to wander. We therefore placed greater value on reflections captured in semi-structured interviews, provided arts-based feedback opportunities involving drawing, colour choice and movement, and tuned in more to the incidental feedback regularly offered by the young people within sessions. Such evaluation methods, however, were not objective and relied on a level of interpretation from facilitators. These interpretations were made through a growing understanding of the language and behaviours these young people were using to highlight their sense of enjoyment, safety, belonging and pride in the moment.

### Choosing Activities

The incidental feedback communicated by the young people in sessions also influenced decisions about session planning and the choice of activities offered. Group leaders were transparent about the purpose of the sessions with participants, who understood that all group members had experienced trauma. They suggested that music and rhythm activities might help to improve their mood and offer a sense of self-control or a feeling of being regulated. We asked explicitly for their feedback on this, and the young people were frequently reminded that they were co-researchers in this project, that their thoughts and ideas about the music or program as a whole was valued and key to the program’s development and success. Rather than being seen through an objective lens as biasing the young people or having a placebo effect, this transparency was essential to the participatory action research approach being adopted (Kemmis et al., 2014).

In addition, believing that the group might be helpful is seen as critical to success in group psychotherapy (Yalom and Leszcz, 2005) and individual therapy (Duncan et al., 2007). That being said, the group leaders’ verbal explanations may not have been very useful, given the complexity of articulating the topic, as described in the rationale. Most importantly, the exploratory and experimental nature of the group enabled these ideas to be examined in an embodied, playful and responsive way throughout the four groups.

To explore the notions of rhythm and regulation, rhythm underpinned each activity implemented in Cycle 1. Chants, props and recordings were used to support structured activities such as the singing of greeting songs, Ti Rakau (a Māori stick game) and beanbag toss, with the sole purpose of deeply embedding rhythm in multi-relational ways (sight, sound, touch). Within hour long sessions, the emphasis on highly structured activities required an intense amount of focus and the young people reported feeling tired and bored. Activities such as songwriting (which might usually offer freedom in style and form) and dance-offs were then included to raise interest and engagement but even so, were limited to modern pop styles with dance beats, which are dependent on strong, steady rhythms. The young people engaged with the creative parts of the songwriting process but displayed some frustration trying to perform their piece within the tight restraints of a rigid rhythm.

Before Cycle 2, the research team reflected on the fatigue this rigidity seemed to cause and incorporated opportunities for more creativity and self-direction within semi-structured activities. Chair drumming for example, allowed the young people to individually explore rhythms along to the beat of a recorded song. It also led to organic opportunities for turn-taking, following and leadership amongst group members, celebrating individual creativity and performance. Noting this enjoyment of less structured activity, and a request for more from the young people, facilitators introduced individual song writing where they could each begin a song from scratch using iPad/GarageBand generated beats and sounds. On reflection, the leap to sessions with far less structure, requiring almost complete self-direction, overwhelmed participants in a different way and none completed a composition, while some reacted with varied levels and displays of frustration, withdrawal and distraction.

By Cycle 3, the young people had led us to understand the need for a balance between both structured and semi-structured activities with a continued emphasis on rhythm. We utilised the benefits of embedding choice and control within a structured “base,” both within whole sessions (choice of order of activities or choice between two activities) and individual activities (tempo, addition of complexity). We also determined that some basic routines (greeting and farewell songs), and strict, shared-rhythm activities (structured drumming) could contribute to greater engagement and success in the more free and creative activities that followed. Drumming to a consistent beat, with the added challenge of “pausing” on a specific count and beanbag toss with physical challenges and an increase in the number of beanbags in play, were activities that were accepted and tolerated by the young people, even with a degree of excitement and enthusiasm. The transition into free dance or group improvisation on a range of instruments with no set beat or rhythm was managed with little to no resistance, and ongoing enthusiasm.

### Building Relationships

During Cycle 1, we noticed that group members regularly spoke over one another and positioned themselves physically in front of each other, in what at first appeared social disregard for those around them. After careful observation across the 10 weeks, we were able to see how this subsided as everyone’s relationship and comfort with the facilitators increased. The facilitators became more aware of each young person’s story, personality, preferences and talents, and were able to acknowledge them in ways that demonstrated how valuable they were in the group. Subsequently, the need to seek attention in more overbearing physical and noisy ways decreased. After reflecting on this change with the research team, individual meetings were instituted from the beginning of Cycle 2. These occurred prior to the commencement of the group itself and incorporated music making activities to prepare the young people for the nature of groups and to better understand their regulatory needs. These meetings supported the facilitators understanding of the young people from the outset and allowed the young people to develop insight into the program they were joining. It also built comfort and rapport and enabled better communication with carers who also had access to the facilitator in the visits, likely contributing to the young people’s trust in facilitators. The impact of this change was so significant that group program duration could confidently be reduced from thirteen weeks to eight weeks and competitive and attention seeking behaviours substantially decreased.

## Discussion and Meta-Reflections

In addition to the learnings that were adopted throughout the cycles of action and reflection that are described above, the research team also reflected on the actions of the group participants (participant, leaders, carers and organisers) at a more propositional level (to draw on Polanyi’s distinctions). Our focus for the meta-reflections was on regulation/dysregulation, because of its prominence as a topic within trauma studies and being the facet of experience that is most often linked to rhythm, as noted in the introduction. Our considerations drew on Siegel’s (1999) conceptualisation of traumatised people having a narrower “window of tolerance” caused by nervous system dysregulation. Research has already shown that a small suite of activities, including rhythm, physical exercise, and mindfulness have seemed to assist some people to return to a state of psychological and physiological arousal (Ogden et al., 2006). By extension of the same theoretical conceptualisation, practicing these kinds of activities should lead to an expanded window of tolerance resulting in less time being spent in either hypoarousal (where the parasympathetic nervous system produces a freeze response) or hyperarousal (where the sympathetic nervous system produces a fight or flight response).

The flaw in this logic, as noted in the introduction, is that many young people are not able to settle into a rhythm, which can therefore lead to further anxiety or withdrawal if encouraged to participate in something that might be beyond their current capability. For example, a child in Cycle 2 was observed to become very frustrated when he could not match the rhythm of the group, and left the group to practice the activity solo, before re-joining the group once he had mastered the rhythm. The same child described how the thundering of the drumsticks on chairs during a rhythm activity was an unpleasant sensory experience and again, removed himself from the group briefly while the facilitators adjusted the activity. He was often observed to be hyperaroused, and therefore the arousing quality of rhythm could easily push him further away from his window of tolerance, rather than closer to it. However, other children in the same group described the same experiences as helpful. One girl who was often observed to be hyperaroused said that she loved chair drumming because “you didn’t have to think.” Another girl in Cycle 1, who also tended toward hypoarousal, similarly commented that “a steady beat helps steady my mind.”

The degree of challenge experienced by each child as they joined in rhythmic activities varied markedly. Sometimes activities that were too easy were experienced as boring and unsuccessful in engaging the young people. For example, in Cycle 1, two young people who often presented with high energy levels reported enjoying a game that involved tossing a small bag of beans in time with a beat, using chanting and name calling to supplement the movement. However, others in the group who had lower energy levels described the beanbag toss as boring. The second group in Cycle 3 struggled most with activities that were too simple and began to explore ways to extend the physical challenge of tasks such as standing in “tree pose” or on a chair whilst tossing the beanbag. This resulted in some group members feeling “happy and settled.”

Change in energy levels were also extremely varied between children whilst engaging in the rhythm activities, again, not always in the same direction. For example, one child in the first group in Cycle 3 would often arrive in a highly energised state and focus hard to participate and then report feeling tired afterward. Another child in the same group often described feeling tired upon arrival and then feeling energised after the rhythm activities and “ready” for creative activities such as song writing. She was also observed to have low arousal levels when feeling anxious, such as when a new carer was watching the program.

Examining the relationship between regulation, rhythm and trauma was further complicated in our research project by the neurodiversity that was apparent in many young people in our groups (6 of the 16 had diagnoses of Autism or ADHD). Although there is no relationship between Autism and trauma, there is debate about whether adverse experiences contribute to or exaggerate ADHD symptomatology (Szymanski et al., 2011). Relatedly, research has shown that the core symptoms of ADHD are part of an Autism diagnosis and that children with Autism might initially be diagnosed as having ADHD (Mayes et al., 2012). These overlaps were useful in reflecting on the diversity apparent in our research groups and led us to reflect on the different language that might be used regarding regulation depending on the diagnostic labels common in each field. For example, discussion of arousal in the autism literature often uses more psychotherapeutically informed terms such as co-regulation and self-regulation (Ting and Weiss, 2017), rather than the constructs of hyper and hypo-arousal that are more common in ADHD and trauma literature.

There were several children in our groups whose ways of connecting through rhythm seemed better understood using the terminology of co-regulation, along with more relational notions such as attachment (Kinniburgh et al., 2017). For example, one child in Cycle 2 seemed to be co-regulating with the facilitator during a particularly difficult day after discovering she would not be able to see her mother in the usual access time. After playing together with the music therapist, her mood seemed to brighten and she said, “Music is good for your brain, like an exercise… but you don’t have to move, you can listen or think.” In the same group, another child seemed to self-regulate by communicating that he needed to take some time out when the group got loud and boisterous. A child from Cycle 3 took this a step further and started using drumming actions at school when he was feeling angry, taking himself outside to a tree he could play on until he felt better. The school went on to purchase a drum pad after liaising with the program facilitators so this could be supported.

**FIGURE 1:**
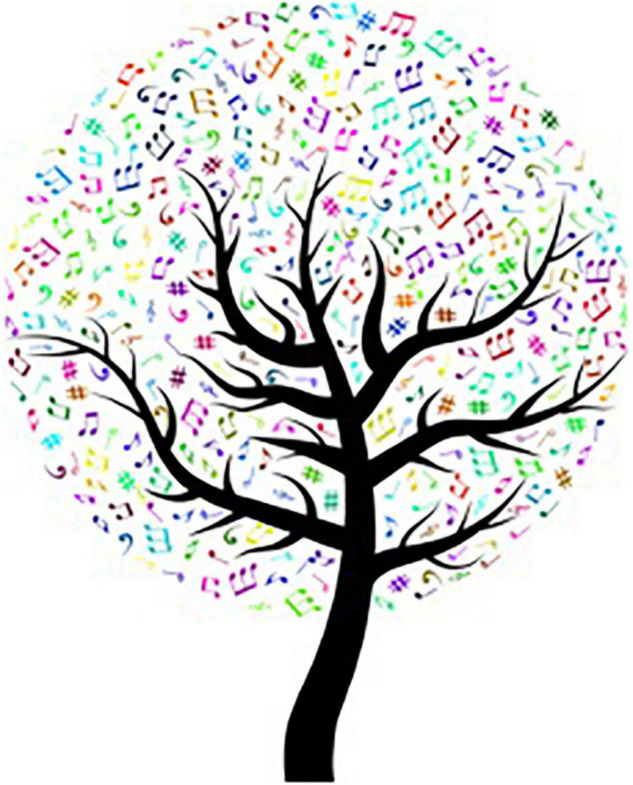
A tree with a dark trunk and expansive branches, its leaves consist of a burst of colourful music notes, clefs, sharps. and flats.

The academic members of our research team undertook critical interpretive review of the literature (McFerran et al., 2020) and further delineated these ideas into uses of rhythm including stabilising (physiological regulation), entraining (co-regulation), exploration (emotion regulation) and performing (self-regulation). Both stabilising and entraining placed a stronger emphasis on the value of the structural components of music, including rhythm, that we were focussing on in the current research. However, we found that all four dimensions were relevant for the diverse young people in different groups in this project and trying to remain focussed only on one or two dimensions did not satisfy or engage the young people. Our meta-reflections on these findings led to the generation of a creative image that was inspired by comments made by some group members (see Figure 1). One child described the different program activities as elements of a tree, and two others bought images of trees into different activities (one drew a family tree, and another introduced a tree pose to bean bag throw). Bringing these together as a metaphor provided us with an arts-based representation of multiple levels of knowing.

The roots and trunk of the tree represented safety in relationships with the facilitators and with other group members which was the core emphasis in stabilising activities The roots of the tree represented each co-contributor, their individual histories, lineages, and experiences, spoken and unspoken. The trunk of the tree illustrated a meeting place between all group members; the young people and facilitators, where safety was the primary focus. In this focus was an acknowledgement by facilitators of shared experiences of trauma between the young people, an acceptance of how each young person presented and a core emphasis on stabilising activities.

The branches of the tree represent invitations and opportunities for making music together in individual or shared rhythms. Sharing music making in this way created potential conditions for co-regulation or self-regulation. As tree branches co-shape one another, so did group members and facilitators; shaping and adjusting activities to levels of individual comfort or challenge, and in group members showing great empathy and acceptance toward one another.

The leaves of the tree represented possibility, dreams, and play. Music/rhythm was an individual and collective compass in this space where young people could explore different ways of being and identities. One young person emerged as a rapper, freestyling with the group, another described playing the guitar as a huge achievement one of which his parent would be proud, and another contextualised their musical identity through family, in sharing songs they would play at home.

All trees change with the seasons, so too did each of the young people (and facilitators). There was always space among the branches, with safer conditions, the young people showed us how they used music and rhythm to share individual truths and experiences, and to feel happy and settled.

## Conclusion

This research aimed to explore the question of how satisfied young people in the out-of-home-care system were with participating in various rhythm-based music therapy activities. We collected a range of data that included verbal and written feedback as well as observing and analysing their creative, playful and behavioural responses and actions. The use of an action research framework enabled a responsive approach where we could make adjustments throughout the process to allow for change and improvement in ways that would help us better answer the question, which was focussed on the satisfaction of the young people in the group. Our intention was to privilege the voices of young people and for them to influence the increasingly neurological approaches favoured in the literature. We wanted to know whether young people’s experiences were congruent with the idea that rhythm-based activities would support them to be able to regulate their emotional reactions and expand their window of tolerance.

The focus of each action and reflection cycle in this study was on trying to understand when rhythm was experienced as helpful by young people by observing their personal, interpersonal and creative behaviours as well as listening to their feedback. In the reflection stages between each cycle we tried to identify whether there were any conditions that might explain when rhythm was helpful – potentially the young person’s age, their unique background experiences, their current home conditions, their ability to regulate their own behaviour, the group membership, and other factors. It became apparent that all these things did converge to influence their experience of the helpfulness of rhythm and of the therapy group generally, but in idiosyncratic ways. This multidimensionality was consistent with statements made by other critical scholars in the field, who suggest avoiding the tendency to try and simplify responses to trauma.

“By de-linking from traditional ways of thinking about trauma, we can reconsider what trauma is in all of its manifestations: specific and murky, lived and remembered, physical and emotional, individual and communal, past and present, named and unnamed, spoken and silenced, fragmented and cohesive, destructive and healing, threatening and empowering.” (p. 14, Gagnon and Novotny, 2020).

This was abundantly apparent when working with the young people in this research who each had their individual stories. As they shared their music with us, we felt privileged to witness a piece of who they were and how they were surviving a world that had been unfair to them. For the young people involved, in some ways what they needed was simple. Some described much-needed relief through moments of restoration and reprieve, such as when one young person said simply, “I could relax because I wasn’t at home.” But we also felt conscious that we should avoid the allure of simplification and saviourism, as Gagnon and Novotny (2020) note. We felt fortunate to be able to offer a group, funded by a university research grant, to children who otherwise might not have access to simple, creative, safe experiences with trusted others. Listening to children’s voices resulted in important findings that represented the significance of groups in ways that quantitative research has failed to capture, likely because of the diversity of individuals and the need for to describe average benefits across all members (for example: Jee et al., 2015; Auslander et al., 2017).

The cycles of action and reflection have enabled us to bring young people’s voices and preferences into the discourse by illustrating how they responded to rhythm-based activities and responding to what they shared of their needs in the context of their unique life stories. They told us that they liked sharing time in music, and that it helped to distract them, to have fun, and to feel safe. These simple experiences should be readily available to all children, but are not easily accessible for those who struggle in school and at home due to challenges in regulating themselves. Although rhythm may not be a panacea in itself, it is an inherent element in most music making experiences and when combined with tempo and form, was prominent in the types of music experiences that the young people responded to. For this reason, we suggest that making music with safe and responsive adults who are able to adjust activities creatively and tailor the structure of groups to meet the needs of the individuals in their particular groups might address the WHO’s Convention on the Rights of the Child (Article 31, Children’s Version) that states: “Every child has the right to rest, relax, play and to take part in cultural and creative activities” (UNICEF, 2020). Some children may need more support than others to be able to access the affordances of rhythm and music and this is where creative, responsive and trustworthy adults have a role to play, both in advocating for funding and providing these opportunities.

## Data Availability Statement

The datasets presented in this article are not readily available because of the small sample size, anonymity may be compromised if data is shared. Requests to access the datasets should be directed to k.mcferran@unimelb.edu.au.

## Ethics Statement

The studies involving human participants were reviewed and approved by The University of Melbourne, Human Research Ethics Committee (ID# 1852103) Developing and piloting a rhythm-based, group music therapy program for children who have experienced trauma. Written informed consent to participate in this study was provided by the participants’ legal guardian/next of kin.

## Author Contributions

KM conceived and led the project, solicited the funding, and wrote the manuscript. KT and ZK co-facilitated the groups, collected the data, and contributed to the manuscript. HS and AC contributed to research design, analysis and the manuscript. All authors contributed to the article and approved the submitted version.

## Conflict of Interest

The authors declare that the research was conducted in the absence of any commercial or financial relationships that could be construed as a potential conflict of interest.

## Publisher’s Note

All claims expressed in this article are solely those of the authors and do not necessarily represent those of their affiliated organizations, or those of the publisher, the editors and the reviewers. Any product that may be evaluated in this article, or claim that may be made by its manufacturer, is not guaranteed or endorsed by the publisher.

## Appendix

Appendix 1: Researcher-created measure of regulation used in Cycle 2.

**Table:** Please tell us how often during today’s session you felt:

	Not at all	Not much	Don’t know	A little bit	A lot
Safe					
Like you belonged					
Free to express yourself					
Proud to have achieved something					
Happy with choices you made					
Regulated – in control of your thoughts & actions					

If you’d like to, let us know WHICH activities helped you to feel these ways and WHAT about the activities you liked?
