# Corrigendum: What young people think about music, rhythm and trauma: An action research study

**DOI:** 10.3389/fpsyg.2022.1060378

**Published:** 2022-11-22

**Authors:** Katrina McFerran, Alex Crooke, Zoe Kalenderidis, Helen Stokes, Kate Teggelove

**Affiliations:** ^1^Faculty of Fine Arts and Music, The University of Melbourne, Melbourne, VIC, Australia; ^2^Melbourne Graduate School of Education, The University of Melbourne, Melbourne, VIC, Australia

**Keywords:** rhythm, trauma, music therapy, action research, co-regulation, arousal

In the published article, there was an error. In Actions and Reflections within the Program, Exploring Structure for the Program, Paragraph 1, the role of one of the authors was not stated. The sentence “Two qualified music therapists co-facilitated groups of 3–5 members, lasting between 8 and 13 weeks,” should be “Two qualified music therapists co-facilitated groups of 3–5 members, lasting between 8 and 13 weeks, supported by a community facilitator who was also one of the researchers.” The corrected paragraph appears below:

There were three cycles of action and reflection across a 12-month period involving a total of 16 young people (see Table 1). Two qualified music therapists co-facilitated groups of 3–5 members, lasting between 8 and 13 weeks, supported by a community facilitator who was also one of the researchers. Group size was intended to include a maximum of 6 young people, but the number of cycles was not pre-determined, except by the 2-year time frame of research funding.

In addition in Actions and Reflections within the Program, Choosing Activities, Paragraph 3 there was an error. The style of music was wrongly characterized as hip-hop but is better described as modern pop styles with dance beats. The corrected paragraph appears below:

To explore the notions of rhythm and regulation, rhythm underpinned each activity implemented in Cycle 1. Chants, props and recordings were used to support structured activities such as the singing of greeting songs, Ti Rakau (a Māori stick game) and beanbag toss, with the sole purpose of deeply embedding rhythm in multi-relational ways (sight, sound, touch). Within hour long sessions, the emphasis on highly structured activities required an intense amount of focus and the young people reported feeling tired and bored. Activities such as songwriting (which might usually offer freedom in style and form) and dance-offs were then included to raise interest and engagement but even so, were limited to modern pop styles with dance beats, which are dependent on strong, steady rhythms. The young people engaged with the creative parts of the songwriting process but displayed some frustration trying to perform their piece within the tight restraints of a rigid rhythm.

The authors apologize for this error and state that this does not change the scientific conclusions of the article in any way. The original article has been updated.

## Publisher's note

All claims expressed in this article are solely those of the authors and do not necessarily represent those of their affiliated organizations, or those of the publisher, the editors and the reviewers. Any product that may be evaluated in this article, or claim that may be made by its manufacturer, is not guaranteed or endorsed by the publisher.

